# Conscious Experience and Episodic Memory: Hippocampus at the Crossroads

**DOI:** 10.3389/fpsyg.2013.00304

**Published:** 2013-05-30

**Authors:** Ralf-Peter Behrendt

**Affiliations:** ^1^Elderly Mental Health Team, Princess Elizabeth HospitalSt Martin, Guernsey, UK

**Keywords:** consciousness, default-mode network, emotional feelings, episodic memory, hallucinations, mental imagery, schizophrenia, θ oscillations

## Abstract

If an instance of conscious experience of the seemingly objective world around us could be regarded as a newly formed event memory, much as an instance of mental imagery has the content of a retrieved event memory, and if, therefore, the stream of conscious experience could be seen as evidence for ongoing formation of event memories that are linked into episodic memory sequences, then unitary conscious experience could be defined as a symbolic representation of the pattern of hippocampal neuronal firing that encodes an event memory – a theoretical stance that may shed light into the mind-body and binding problems in consciousness research. Exceedingly detailed symbols that describe patterns of activity rapidly self-organizing, at each cycle of the θ rhythm, in the hippocampus are instances of unitary conscious experience that jointly constitute the stream of consciousness. Integrating object information (derived from the ventral visual stream and orbitofrontal cortex) with contextual emotional information (from the anterior insula) and spatial environmental information (from the dorsal visual stream), the hippocampus rapidly forms event codes that have the informational content of objects embedded in an emotional and spatiotemporally extending context. Event codes, formed in the CA3-dentate network for the purpose of their memorization, are not only contextualized but also allocentric representations, similarly to conscious experiences of events and objects situated in a seemingly objective and observer-independent framework of phenomenal space and time. Conscious perception, creating the spatially and temporally extending world that we perceive around us, is likely to be evolutionarily related to more fleeting and seemingly internal forms of conscious experience, such as autobiographical memory recall, mental imagery, including goal anticipation, and to other forms of externalized conscious experience, namely dreaming and hallucinations; and evidence pointing to an important contribution of the hippocampus to these conscious phenomena will be reviewed.

## Introduction

Cognitive-neuroscience theories of consciousness suggest that consciousness arises from stable patterns of activation in widely distributed brain areas. Synchrony and resonance between distributed networks that are activated above threshold would “bind” different perceptual features into a unitary conscious experience (reviewed in Atkinson et al., 2000). Conscious experience, as opposed to unconscious processing, of external stimuli has indeed been associated with synchronized neuronal activity across widely distributed neocortical areas, including parietal areas (Palva et al., 2005; Melloni et al., 2007). The question remains as to how the information content of consciousness can be derived from distributed neocortical modules that are specialized for processing stimuli in relation to functions of attention, working memory, and action disposition or preparation; and that, in posterior parietal areas, encode stimuli in egocentric, action-oriented frames of reference (Colby and Goldberg, 1999; Andersen and Buneo, 2002), whereas, consciously, stimuli are perceived in an allocentric frame of reference, that is, in the external context of space and time.

The hippocampus is concerned with continuously recording attended experience, forming memories of events as they happen in their spatiotemporal context (Burgess et al., 2001; Morris et al., 2003; Kubik et al., 2007) and linking event codes into episodic memories, that is, “sequences of events that unfold over time and space” (Eichenbaum and Fortin, 2005). The hippocampus also plays a critical role in episodic memory recall – a function that is closely related to mental imagery or internal simulation of goals or outcomes (Addis et al., 2007; Schacter and Addis, 2007). If we were to argue that episodic memory formation is similarly closely related to conscious experience of the seemingly external world, then the neural mechanism underlying episodic memory functions should be closely related, if not identical, to the neural mechanism underpinning consciousness. The hippocampus would “bind” consciousness in as much as it is acting to bind and encode episodic (declarative) memories. The formation of event memories involves the integration of object information and spatiotemporal information into a unique representation through processes of pattern separation and completion in the hippocampus, especially the dentate gyrus and hippocampal region CA3 (Kesner, 2007; Rolls, 2007). Neocortical processes representing features of objects and their external context converge onto a single pattern of neuronal activity rapidly emerging in region CA3. It is proposed that patterns of activity forming in the autoassociation network of CA3 at each cycle of the local θ rhythm encode attended experience and have the informational content of objects or events embedded in an allocentric context – the informational content of unitary conscious experience.

Moreover, the hippocampus forms a representation of the animal’s environmental context, that is, the animal’s place within its environment, and plays a key role in navigation across spatial contingencies of the environment (O’Keefe, 1976; Brun et al., 2002; Anderson and Jeffery, 2003). In many species, places occupied by the animal are characterized by visual landmarks; and what we experience consciously may be an epiphenomenon of a process that maps view-dependent allocentric information about landmarks onto view-independent place representations. Landmarks situated in a spatial context could have evolved into situated objects that are linked to emotional behavior modes, while the spatial context, in which objects are embedded or events occur, evolved into a more general framework of space and time; consistent with the proposed derivation of phenomenal time form phenomenal space (Boroditsky, 2000). By recurrently forming representations of landmarks or objects that are situated in a spatiotemporal context, the hippocampus creates phenomenal space and time, consistent with philosophical insights that the framework of space and time that appears to surround us is not an objective reality but a fundamentally subjective creation (Schopenhauer, 1844/1966).

Reviewing theories of consciousness, Zeman (2001) concluded that “no theory of this kind satisfactorily explains what phenomenal awareness contributes to information processing” (p. 1281). Contemporary theories of consciousness tend to assume that “consciousness makes a difference” and “plays an important, more or less continuous role in directing our waking behavior” (Zeman, 2001, p. 1282). However, psychophysical evidence suggests that “consciousness arises too late to perform the kinds of function which these theories envisage,” so that, perhaps, “rather than guiding action from moment to moment, consciousness serves its biological purpose on a longer, more reflective, time scale” (Zeman, 2001, p. 1281). It was Chalmers’ (1996) proposal that, because “consciousness involves a separate class of non-physical properties,” “subjective qualities of experience” cannot “make a difference to the physical trajectory of human behavior” (as reviewed in Zeman, 2001, p. 1284). Indeed, from our first-person perspective, we have no access to the physical trajectory of behavior or to mechanisms executing behavior, as they are part of a realm beyond our conscious experience (Behrendt, 2007). The behavior executed in the physical realm is not what we experience consciously. The behavior of which we are aware is but an *awareness of behavior* – evidence perhaps for a reflective or mnemonic process, or evidence for a process that dynamically characterizes our current *situation* (or position) in a multidimensional spatiotemporal state space and that allows the organism to *unconsciously* respond to stimuli in a *situationally appropriate* manner.

## Convergence of Dorsal and Ventral Visual Processing Streams

Object-related information, processed by the ventral visual stream, and action-related spatial information, processed by the dorsal visual stream, converge in the hippocampal formation in the medial temporal cortex. The ventral visual stream courses via the occipitotemporal cortex, the anterior lateral temporal cortex (a unimodal association area representing visual objects, as well as social cues, in terms of conjunctions of qualities or features), the perirhinal cortex [an area that processes novelty, familiarity, or recency of objects (Brown and Aggleton, 2001; Pihlajamäki et al., 2004; Eichenbaum et al., 2007)], and the *lateral* entorhinal cortex [which, too, plays an important role in recognition memory, including the recognition of individual conspecifics (Petrulis et al., 2005)] to the anterior hippocampus (head of the hippocampus) (Kahn et al., 2008; Kravitz et al., 2011). The dorsal visual stream runs via the occipitoparietal cortex to the lateral posterior parietal cortex (superior and inferior parietal lobules), where salient visual stimuli are encoded in frames of reference relative to parts of the body and the eye (egocentric reference frames) for the purpose of guiding particular types of motor acts, forming “action-oriented spatial representations” (Colby and Goldberg, 1999; Shadlen and Newsome, 2001; Andersen and Buneo, 2002). The dorsal visual stream trifurcates into the parietoprefrontal pathway (connecting the inferior parietal lobule with the dorsolateral prefrontal cortex and subserving spatial working memory), the parietopremotor pathway (connecting the superior parietal lobule with dorsal premotor regions and subserving unconscious visually guided actions), and the “parieto-medial temporal pathway” (playing a crucial role in spatial navigation) (Kravitz et al., 2011). The “parieto-medial temporal pathway” courses from the inferior parietal lobule directly to structures in the medial temporal lobe; and it courses indirectly via medial parietal lobe structures to the medial temporal lobe (Kravitz et al., 2011).

The *rostral* portion of the inferior parietal lobule is specialized for guiding manual actions in *peripersonal* *space* (reaching, grasping, holding, placing) and projects to the ventral premotor cortex, which encodes object-guided manual actions. It is the *caudal* portion of the inferior parietal lobule that is specialized for processing *distal* *space* information necessary for guiding navigational behavior; and it is this region that projects directly to structures in the medial temporal lobe, including hippocampal region CA1 and the posterior parahippocampal cortex, as well as to regions of the medial parietal lobe, including the retrosplenial cortex and posterior cingulate cortex (reviewed in Kravitz et al., 2011). The retrosplenial and posterior cingulate cortices, receiving spatial information processed in lateral posterior parietal regions (especially the inferior parietal lobule), project to the posterior parahippocampal cortex and the entorhinal cortex in the medial temporal lobe. The posterior parahippocampal cortex, in turn, projects via the *medial* entorhinal cortex to the posterior hippocampus (body of the hippocampus) (reviewed in Mohedano-Moriano et al., 2007). In humans, the inferior parietal lobule, retrosplenial cortex, posterior cingulate cortex, posterior parahippocampal cortex, and posterior hippocampus are functionally connected with each other (as shown by correlations between spontaneous BOLD signal fluctuations in these regions at rest) (Kahn et al., 2008).

Medial parietal areas represent spatial locations of landmarks or salient objects that are visible from the animal’s current position. While the lateral posterior parietal cortex encodes visual stimuli in retinocentric and other egocentric coordinates, the retrosplenial cortex, adjacent precuneus, and portions of the posterior cingulate cortex in the medial parietal lobe encode navigationally relevant landmarks in egocentric, head-centered coordinates (reflecting the animal’s head direction) or in allocentric coordinates (“world coordinates,” that is, coordinates that are “fixed” to the external world) (Dean and Platt, 2006; Byrne et al., 2007; Kravitz et al., 2011). The retrosplenial cortex and posterior cingulate cortex contribute to the transformation of egocentric representations into allocentric representations, which are found especially in the medial temporal lobe (Byrne et al., 2007). The posterior parahippocampal cortex, receiving information from the retrosplenial cortex and posterior cingulate cortex, encodes landmarks and their spatial arrangement, or places them within a spatial context (reviewed in Burgess et al., 2001; Pihlajamäki et al., 2004; Kravitz et al., 2011). The hippocampus, using “pattern completion” and “attractor dynamics,” then embeds landmarks and objects in an allocentric representation of the environment (Byrne et al., 2007).

## Default-Mode Network

The inferior parietal lobule and posteromedial parietal areas, namely the posterior cingulate cortex, retrosplenial cortex, and precuneus, belong to the “default-mode network” (which also includes ventromedial prefrontal cortex, mid-dorsolateral prefrontal cortex, anterior temporal cortex), which supports mental processes during the conscious resting state (“default mode”) and which is deactivated (or attenuated) during the performance of goal-directed actions or cognitive tasks (Raichle et al., 2001; Buckner et al., 2008). Default-mode network activity supports broad information gathering about the world (broad attention) during exploratory monitoring and monitoring for unexpected events, enabling the detection, for example, of predators without the need to intentionally allocate attentional resources (Gusnard et al., 2001; Raichle et al., 2001; Buckner et al., 2008). The inferior parietal lobule, especially in the right hemisphere, subserves the ability to detect behaviorally relevant stimuli that are unrelated to the present task; and a lesion in this region causes unilateral visuospatial neglect (usually left-sided) (Halligan et al., 2003; Pessoa et al., 2003). The posterior cingulate cortex and adjacent precuneus in the medial parietal lobe represent the visual periphery and support broad monitoring of the external environment. Damage to the medial parietal lobe, including the posterior cingulate and precuneus, causes an inability to perceive objects outside the focus of attention (“simultanagnosia”). Broad monitoring of the external world, allowing the animal to characterize the situation it faces, may be the evolutionarily primary function of the default-mode network.

A broad low-level focus of attention (relaxed attention) is similarly adopted in association with internal cognition (introspection). The default-mode network, when active in states of rest, can gather information within the “internal environment” in order to assess its salience for the individual, similarly to how it surveys the external environment (Gusnard et al., 2001; Raichle et al., 2001; Buckner et al., 2008). Related to this function is the monitoring of the self. The precuneus, especially its anterior portion, is a central node in the network activated during mental imagery and “internal mentation.” The precuneus and the wider medial prefrontal-mid-parietal network also play an important role in conscious self-awareness, self-reflection, and cognition about self-representations (Cavanna and Trimble, 2006). The self can be bolstered by imagery of others’ appreciative attitudes toward ourselves, but the self is primarily a distillate of our position in, or relatedness to, the social world around us. In social situations, the default-mode network enables us to consider the thoughts and perspectives of other people (Buckner et al., 2008) and, perhaps especially, to monitor others’ attitudes toward ourselves and toward the values or norms by which we define ourselves.

Activity in regions of the default-mode network correlates with people’s proclivity to mind-wander and generate spontaneous thoughts and mental images during periods of reduced central executive demand (Mason et al., 2007). Evidently, “conscious information in the form of mental images and spontaneous thoughts” can be manipulated “for problem solving and planning” (Cavanna and Trimble, 2006, p. 577). In the “absence of external task direction,” regions within the default-mode network “facilitate flexible self-relevant mental explorations – simulations – that provide a means to anticipate and evaluate upcoming events” (Buckner et al., 2008, p. 2). Activity in posteromedial parietal areas, such as the posterior cingulate cortex and retrosplenial cortex, increases not only during the conscious resting state but also during lapses in focused attention (Weissman et al., 2006). Attention lapses during task performance, reflecting transient shifts from task-related network to default-mode network activity, can be harnessed for outcome simulation, goal setting, and decision making. The precuneus is activated in association with imagery of action outcomes; and its connection with rostral anterior cingulate cortex suggests that it makes an important contribution to the guidance of voluntary behavior by outcomes or goals anticipated in imagery (Krieghoff et al., 2009).

Parietal regions that are part of the default-mode network are functionally connected (showing correlated slow BOLD signal fluctuations) with the posterior parahippocampal cortex and hippocampal formation (hippocampus and entorhinal cortex) (Greicius et al., 2003, 2004; Vincent et al., 2006; Kahn et al., 2008), suggesting that “the default-mode network is closely involved in episodic memory processing” (Greicius et al., 2004, p. 4641). The precuneus, posterior cingulate, retrosplenial cortex, and inferior parietal lobule are indeed activated during the recall of visuospatial or autobiographic memories (Cavanna and Trimble, 2006; Vincent et al., 2006; Takashima et al., 2007), confirming that “recollection of personally relevant memories is a component of the cognitive content of the default state” (Vincent et al., 2006, p. 3528). Conscious (vivid and context-rich) recall of autobiographical memories was shown to be associated not only with activation of the posterior cingulate and precuneus but also with that of the left hippocampus (Gilboa et al., 2004). The hippocampus, a key structure of the episodic memory system, may bind experiential features during the retrieval and conscious elaboration of past autobiographical events (Addis et al., 2007). Imagery of future events of personal significance similarly activates the hippocampus, as well as medial parietal regions, leading to the proposition that the ability of the hippocampus to extract and recombine elements of past experiences can be utilized for purposes of simulation of future events, decision making, and prospective guidance of behavior (Addis et al., 2007; Schacter and Addis, 2007).

Thus, default-mode network activity “involves retrieval and manipulation of past events, both personal and general, in an effort to solve problems and develop future plans” (Greicius et al., 2003, p. 257); and even the internal generation of “action intentions draws especially heavily on the prospective memory network” (Krieghoff et al., 2009). The episodic memory system, including hippocampus and default-mode network, extracts and recombines elements of previous experience so as to simulate outcomes and enable “evaluation regarding future approach or avoidance of similar scenarios”; outcome simulation, based on recombination of episodic memories, “enables one to consider whether a particular situation would be approached or avoided if encountered” (Addis et al., 2007). For example, simulation of future outcomes, triggered by social cues emanating from the present situation, is accompanied by fleeting experiences, in imagery, of social situations in which one is appreciated or rejected by others. Such simulations are “unconscious” in the sense that they are not readily acknowledged to oneself or others. Simulation of social experience that involves criticism or rejection, based on past experience, has the effect of producing social inhibition or avoidance. It is interesting to note that, in patients with schizophrenia, who tend to be sensitive to social rejection and are often socially inhibited, default-mode network activity is increased in association with more severe hallucinations and delusions (Garrity et al., 2007) and greater “emotional awareness for others” (Harrison et al., 2007).

In summary, the default-mode network may support characterization of the present or an anticipated situation by providing relevant cues to the hippocampus, where newly formed or recombined event memories may channel into a process of self-localization with respect to a multidimensional state space that captures contingencies of the external world, contingencies that, in primates and especially humans, extend well beyond the topographical layout and temporal cycles of the external physical environment, to include social structures and norms organized on multiple levels. Consciousness may be evidence for episodic memory functions serving such self-localization, which, time and again, sets the framework within which the organism unconsciously responds to *relevant* external stimuli and performs appropriate habitual tasks. The default-mode network is in dynamic competition with a neocortical network that subserves focused attention and the translation of relevant stimuli into actions during task performance (Greicius et al., 2003; Buckner et al., 2008). The task-related (task-positive) network, competitively interacting with the default-mode network, includes dorsolateral and ventral prefrontal regions, lateral parietal regions (intraparietal sulcus/posterior parietal cortex), and a middle temporal region, as well as the dorsal anterior cingulate cortex (Fox et al., 2005; Boly et al., 2007). A transient shift from default-mode network activity to task-positive network activity, as broad attention shifts to focused attention, facilitates the perception of task-related stimuli during task performance (Weissman et al., 2006; Boly et al., 2007). Perception of, and responding to, task-relevant stimuli may occur outside consciousness, but it may well be the case that, especially in humans, even the performance of seemingly routine activities requires frequent switching between the two networks, giving rise to an impression of continuous consciousness accompanying these activities.

## Episodic Memory

Posterior parietal regions that form part of the dorsal visual stream send information via the parahippocampal cortex and medial entorhinal cortex to the posterior hippocampus (corresponding to the dorsal hippocampus in rodents) and thus influence the ongoing recording of spatial experience by the posterior hippocampus. Unimodal association areas of the neocortex that represent qualities or features of objects provide information to the perirhinal cortex, which, in turn, is connected via the lateral entorhinal area with the anterior hippocampus (corresponding to the ventral hippocampus in rodents). Perirhinal and lateral entorhinal areas are interconnected with the amygdala and orbitofrontal cortex. Information about different rewards and punishers, represented in the amygdala and orbitofrontal cortex, reaches the anterior hippocampus via the lateral entorhinal cortex. The hippocampus associates information about objects, their meaning, and their context, conveyed by inputs from the lateral and medial entorhinal areas, to form relational memory representations (reviewed in Eichenbaum et al., 2007). Hippocampal activity encodes events as conjunctions of items and their contextual associations and links these events sequentially to form episodic memories (Fortin et al., 2004; Eichenbaum and Fortin, 2005). The hippocampus is engaged in continuous recording of attended experience, recording arbitrary “events” as they occur in their spatiotemporal context (Burgess et al., 2001; Kubik et al., 2007). Having the “capacity to remember the flow of events,” the hippocampus can “replay” these memories “as a sequence of events and where they occurred in a previous experience” (Ergorul and Eichenbaum, 2004, p. 403). Neuroimaging research in humans supports the notion that the hippocampus plays an important role in encoding and retrieval of episodic memories, including autobiographical memories (Zeineh et al., 2003; Gilboa et al., 2004; Eldridge et al., 2005; Prince et al., 2005), and in the prediction of future events on the basis of a single prior experience of a sequence of events (an episode) (Kumaran and Maguire, 2007). The left hippocampus, in humans, represents sequential aspects of episodic experiences and temporal aspects of autobiographical memory, whereas the right hippocampus in humans plays a greater role in spatial processing.

Within the hippocampus, region CA3, receiving inputs from the entorhinal cortex, rapidly encodes complex memory representations involving object information, reward information, and contextual information about the “space out there” (allocentric space), whereby the anterior (ventral) hippocampus is preferentially concerned with non-spatial (object- and reward-related) information, while the posterior (dorsal) hippocampus preferentially processes spatial or contextual information (Rolls and Xiang, 2005; Rolls et al., 2005; Ross and Eichenbaum, 2006; Kesner, 2007; Kubik et al., 2007; Rolls, 2007). CA2 is similar to CA3 in many ways, but may play a special role in forming memories of social encounters (in rodents) (Young et al., 2006). Layer II of the entorhinal cortex projects (via the perforant path) to the dentate gyrus (granule cell layer) and regions CA3 and CA2. The dentate gyrus (granule cell layer) sends axons (mossy fibers) to CA3 (but less to CA2); and backprojections from CA3 return to the dentate gyrus (making synaptic connections with mossy cells). Pyramidal neurons in region CA3 are extensively interconnected via recurrent axon collaterals (“autoassociative” connections) that terminate in modifiable synapses. The high degree of internal connectivity and effective synaptic plasticity in CA3 enable the rapid encoding of a unique identifying pattern representing “arbitrary associations” between information from the temporal cortex regarding the identity of an object and information from the parietal cortex regarding the location of an object (reviewed in Morris et al., 2003; Kesner, 2007; Rolls, 2007). The neural network of the dentate gyrus, projecting via mossy fibers to CA3, implements a “pattern separation” process, serving the creation, in CA3, of “orthogonal pattern representations” of the environment (Kesner, 2007). As event memories are sequentially encoded within the autoassociative network of CA3, backprojections from CA3 pyramidal cells provide feedback to the dentate gyrus, where the modification of synaptic connections between CA3 backprojections and mossy cells may link one event memory with the next in a sequence (Lisman et al., 2005).

Unique identifying patterns formed in hippocampal region CA3 may serve an indexing function (Morris et al., 2003), that is, representations of events formed and stored in CA3 “can be used to reactivate the content of the memory via the return projections to the neocortex” (p. 1494), but it is also possible that event codes formed in CA3 may “reflect the content or context of the event itself in some way” (Burgess et al., 2001, p. 1495). Hippocampal region CA1 receives input from CA3 (and CA2) via Schaffer’s collaterals and links several firing patterns in CA3 to form a “whole scene” memory, a representation of the scene toward which the animal orients (Robertson et al., 1998; Rolls, 2007). CA1 also receives direct input from layer III of the entorhinal cortex (again via the perforant path). Location-specific information that reaches CA1 directly from the entorhinal cortex supports the encoding, by ensemble firing in CA1, of the animal’s location within its environment (Brun et al., 2002; Anderson and Jeffery, 2003; Morris et al., 2003; Kesner, 2007; Kubik et al., 2007). Ensemble activity in CA3, but not in CA1, is sensitive to the *content* of a scene or place in the environment; CA3 supports the retrieval of memories about where an objects is in a scene (Robertson et al., 1998; Rolls et al., 1998) or where a reward could be found in the environment (Brun et al., 2002). When the animal recalls prior experience during navigational decision making, neuronal ensemble firing in CA3 transiently reconstructs a representation of a location ahead of the animal (“non-local representation”) (Johnson and Redish, 2007), which then entrains the CA1 network (involving increased γ-frequency synchronization between CA3 and CA1) (Montgomery and Buzsáki, 2007). Retrieval of a previously formed pattern encoded in CA3 (representing an original event) is initiated by direct entorhinal inputs (from layer II via the perforant path to CA3) representing a partial retrieval cue (fragments of a previous input pattern) (reviewed in Lisman et al., 2005; Kesner, 2007; Rolls, 2007). The proposition is that the sequential event codes formed in CA3, possibly reflecting the content and context of objects or events, underlies unitary conscious experience of objects and events embedded in a spatio-temporally extending world. What is consciously experienced may be the informational content of CA3 neuronal ensembles rapidly forming through attractor dynamics.

Pyramidal cells in the hippocampus fire synchronously at γ frequencies. γ Oscillations in local field potentials recorded from the hippocampus reflect rhythmic inhibitory postsynaptic currents generated in pyramidal cells by parvalbumin-expressing basket cells (GABAergic interneurons that synapse onto somata and initial axonal segments of pyramidal cells) (reviewed in Buzsáki, 1996; Montgomery and Buzsáki, 2007). During exploratory locomotion, behavioral preparedness, attentiveness to stimuli, and REM sleep, γ oscillatory activity in the hippocampus (including the dentate gyrus) and entorhinal cortex is modulated by θ oscillations (which are controlled by cholinergic and GABAergic input from the medial septum) (Chrobak and Buzsáki, 1998; Hasselmo, 2006). θ Oscillations in the hippocampus enhance memory encoding and ensure continuous gathering of information about the environment (reviewed in Buzsáki, 1996; Vertes, 2005). While synchronous γ-rhythmic activity of CA3 pyramidal neurons that is tuned to the θ rhythm may be necessary for the temporary storage of sequences of event memories, θ-modulated neuronal assembly firing in CA1 encodes the animal’s location and the track along which it navigates (reviewed in Buzsáki and Draguhn, 2004; Buzsáki, 2005; Shapiro and Ferbinteanu, 2006). The learning of sequential items in an episodic memory task involves θ oscillations, in the same way that θ is required for the coding of sequential places along a track. θ Periods are necessary for tying together in time neuronal assemblies representing sequential events whatever their nature (Buzsáki, 2005). θ Rhythm, which can also be found in structures around the Papez circuit (Vertes et al., 2004) as well as in neocortical regions, may coordinate neuronal activity in hippocampus, medial temporal and medial parietal regions, and even lateral neocortical regions, during navigational tasks (Caplan et al., 2003) or tasks involving the recall of episodic memories (reviewed in Kahana, 2006; Byrne et al., 2007).

VanRullen and Koch (2003) argued that θ oscillations, acting as a carrier for γ oscillations, provide the context for conscious memory recall, whereas α oscillations would provide the context for conscious perceptual experiences, which would be consistent with psychophysical findings indicative of the discrete nature of conscious perception. Recall of episodic memories in imagery may not be too different from conscious perception, in that conscious perception is always also a recall or recognition of what has been perceived before. The difference between memory recollection and perception, insofar as these are considered to be conscious phenomena, may boil down to the difference between fleeting experience of seemingly private events and more substantial awareness of an external world, which is really also a fundamentally private and subjective experience (Schopenhauer, 1844/1966; Behrendt, 2007). Given that perception of seemingly external events and imagery of seemingly internal events are, on a deeper level, phenomenologically identical, and are, in any event, likely to be evolutionarily closely related, they should be attributable to the same process, namely self-organizing patterns emerging at each wave of the θ rhythm in the autoassociation network CA3. Unique patterns that form in CA3, due to recurrent attractor dynamics, are event codes that capture arbitrary associations between objects and their context and that, in a dimension orthogonal to the physical reality of such processes, manifest as discrete elements of the stream of consciousness. Consciousness is a moment-to-moment reflection of self-organizing pattern formation, recurring at θ rate, in CA3. Whilst these patterns do influence behavior, consciousness – the flow of symbols ascribed to the sequence of event codes – does not. The consciously experienced world, including phenomenal space and time, is orthogonal to, and thus inconsequential for, the physical world, as recognized by Chalmers (1996).

## Emotional Feelings

Olfactory system and hippocampus evolved from a common system concerned with sensing diffusible molecules in the environment (exteroception) (reviewed in Lathe, 2001). The hippocampus plays a critical role in navigation, and the same could be said, perhaps, about the structure from which the hippocampus evolved. A primitive form of allocentric information, in accordance with which the organism’s place preference and place avoidance behavior could be controlled, may have derived from the chemical composition of the environment. As brain ventricles that were originally exposed to the external milieu closed in the course of phylogenesis, and as, therefore, the hippocampus lost its exteroceptive function, allocentric information came to be sourced from visual or auditory modalities, instead, while the olfactory system remained responsive to the external chemical milieu, although the hippocampus (especially the ventral or anterior hippocampus in rodents or primates, respectively) continues to process olfactory information, too (reviewed in Bannerman et al., 2004). As the hippocampus ceased to be exposed to the external chemical milieu, and as navigation became landmark-based, the hippocampus took on a new function: that of “sensing” the hormonal and metabolic milieu of the organism (enteroception) (Lathe, 2001). Lying alongside the cerebral ventricles and the vascular choroid plexus, the hippocampus is accessible to hormones and metabolites present in the cerebrospinal fluid or circulating in the blood stream. Hippocampal function is indeed modulated by a great variety of hormones and metabolites, which Lathe (2001) thought enables the hippocampus to “sense” internal states such as hunger, thirst, fatigue, stress, fever, and malaise, “in much the same way as the olfactory system can sense external substances (exteroception)” (p. 217).

Events can occur, and objects can be found, in a context that is not only spatial and temporal in nature but also emotional. Consciously experienced, that is, situated or situationally embedded, events or objects can be associated with emotional value. Sensing internal hormones and metabolites and representing the organism’s internal milieu, the hippocampus can use enteroceptive information for the formation of emotional memory associations (Lathe, 2001). Not only internal states of hunger or thirst but also states of pain can be used as unconditioned stimuli for associative learning. The hippocampus makes a contribution to pain responsiveness and pain awareness (reviewed in Lathe, 2001), which may be important for learning to avoid situations in which the likelihood of noxious stimulation is high. Indeed, the rodent ventral hippocampus plays an important role in avoidance learning and anxiety-related behaviors (McNaughton and Wickens, 2003; Bannerman et al., 2004; McNaughton and Corr, 2004; McNaughton, 2006), and the same can be said about the human hippocampus (Klein et al., 2007). By embedding events or objects in an emotional context, the hippocampus would draw on its enteroceptive function and ensure that place preference, place avoidance, and more sophisticated navigational behaviors (and derived complex appetitive behaviors) are sensitive and appropriate to the organism’s physiological needs, including its needs for rewards (for stimuli that are associated with satisfaction of hunger or thirst, for example) and safety (that is, the absence of noxious stimulation). Ultimately, a key function of the hippocampus may be to move the organism from a situation of need or from an aversive or anxiogenic situation to a situation in which rewards can be found and in which the risk of harm (or punishment) is low; and the hippocampus may have done so, early in its phylogenetic history, in accordance with the chemical composition of the environment, and it does so now in accordance with spatial and temporal contingencies inherent in the external physical world.

Emotional feelings were proposed to reflect the organism’s visceral and somatic state and to be represented in viscerosensory and somatosensory cortices (Damasio, 1997, 2001; Damasio et al., 2000). The insular cortex, in particular, was implicated in conscious emotional experience (Damasio, 1997, 2001; Singer, 2007). The anterior agranular insula – processing visceral and autonomic information as well as somatic and pain stimuli – is activated in association with conscious experiences of pain (Rainville et al., 1997; Ploghaus et al., 2003; Wager et al., 2004; Boly et al., 2007), fear (Bell et al., 1999), suspicion (Winston et al., 2002), anger, happiness, sadness, disgust, hunger, thirst, as well as reward expectation, and drug craving (London et al., 2000; Kesner and Gilbert, 2007; Luhmann, 2009; Naqvi and Bechara, 2009). The anterior insula may translate information concerning the organism’s bodily state into motivational functions and decision making biases (Contreras et al., 2007; Luhmann, 2009; Naqvi and Bechara, 2009). The anterior agranular insula is reciprocally connected with the ventromedial prefrontal cortex. The ventromedial prefrontal cortex, projecting heavily to hypothalamic and brainstem centers, induces visceral, autonomic, and endocrine alterations that jointly amount to a “somatic emotional response” (Damasio, 1997) and, owing to its place in the default-mode network, provides “emotional biasing signals” to the simulation of the external and internal world (Raichle et al., 2001). Moreover, the anterior agranular insula is in a position to control emotional aspects of the simulated external or internal world via direct projections to the lateral entorhinal cortex. Information from insular and parainsular cortices is forwarded via the lateral entorhinal cortex to the anterior hippocampus [similarly to olfactory information and information processed by the perirhinal cortex (reviewed in Mohedano-Moriano et al., 2007)], enabling representations of the organism’s bodily state to modulate emotional aspects of event memories and, hence, the emotional context of conscious experience, while, at the same time, neocortical processing converging onto the posterior hippocampus modulates the spatiotemporal context of consciousness.

Affective information that is taken into account when a representation of the animal’s current location or situation is translated into an appropriate mode of exploratory, emotional, or instrumental behavior can be derived not only from the physiological state of the organism but also from salient external stimuli. The basolateral complex of the amygdala, responding to motivationally salient or emotionally arousing stimuli, modulates the formation, by the hippocampus, of emotional memories, such as the memory for the environmental context surrounding an exposure to an aversive stimulus (contextual fear memory) (McGaugh et al., 1996). The basolateral amygdalar complex projects directly to hippocampal regions CA3 and CA2. Moreover, the basolateral amygdala modulates sensory information that reaches the dentate-CA3 network of the anterior hippocampus by way of, firstly, projections to the anterior lateral temporal cortex (Amaral, 2002; Compton, 2003) [which, as part of the ventral visual stream, processes object-related information – as well as, in humans, visual and non-visual information about social and emotional cues – and forwards this information via perirhinal and lateral entorhinal cortices to the anterior hippocampus (Kahn et al., 2008)] and, secondly, projections to perirhinal and entorhinal cortices (Rosenkranz and Johnston, 2006; Bauer et al., 2007). Affect-related information that impacts on episodic memory formation includes information about rewards or punishers represented by the amygdala and orbitofrontal cortex, information that reaches the anterior hippocampus via the lateral entorhinal cortex (and that, thereby, influences the formation of “arbitrary associations” between reward or punishment information and information about the “space out there”) (Rolls and Xiang, 2005).

## Dreams

Dreams associated with REM (rapid-eye-movement) sleep and conscious perception and hallucinations during wakefulness are phenomenologically closely related (Behrendt, 2007); and similar, if not identical, mechanisms should underpin these varieties of conscious experience. In both, REM sleep and wakefulness, conscious experience occurs in association with high-frequency, low-amplitude electroencephalographic activity in the neocortex (“desynchronization”) (Llinás and Paré, 1991). Both, REM sleep and waking, are also associated with θ oscillations in the hippocampus. θ Rhythm promotes synchronization of γ oscillatory activity in the hippocampus and entorhinal cortex during both, REM sleep and exploratory or attentive behavior (reviewed in Buzsáki, 1996). In the rat hippocampus, sequences of patterned neuronal ensemble firing that are modulated by θ rhythm may reflect experiences during wakefulness; and their reproduction (corresponding to a reactivation of episodic memory traces) during REM sleep may form the content of dream states (Louie and Wilson, 2001). In particular, pattern formation in CA3 may be responsible for dream imagery. In the rat hippocampus, θ and γ oscillations are highly synchronized, during REM sleep, between dentate gyrus and CA3 (while γ coherence between CA3 and CA1 is reduced); and it was thought that this synchronization may reflect the processing, during REM sleep, of event memories (involving pattern separation and recombination of memory codes) (Montgomery et al., 2008). It appears that, when dreaming, new event codes – reflecting arbitrary associations of objects, events, and their spatiotemporal and emotional context – are formed (at each θ cycle) from fragments of previous experiences; but these codes are generally not retained. Dream memorization in humans was associated with increased functional connectivity (evidenced by coherence between local oscillatory activities, especially in the low-frequency range), during sleep, between rhinal cortices and hippocampus and between anterior and posterior parts of the hippocampus (Fell et al., 2006). While hippocampus and rhinal cortices are actively involved in dreaming, the default-mode network is not. The precuneus and adjacent regions in the posterior medial parietal cortex show marked deactivation during REM sleep, despite extensive evidence pointing to an active contribution of the precuneus to mental imagery and conscious experiences of other kinds (reviewed in Cavanna and Trimble, 2006).

Obviously, there is no formation of episodic memories when there is no consciousness (such as in states of deep sleep or general anesthesia). Conversely, when there is consciousness, such as in dream states (or even in daydreams and states of inner mentation), there must be episodic memory *formation*, too, albeit not necessarily followed by memory retention and consolidation. It is claimed here that it is the ongoing process of recurrent formation or registration of episodic memories, *not their consolidation or retention*, that is necessarily linked with consciousness. It could be objected that there is no obvious abolishment of consciousness in patients with severe amnesia, however, in patients with amnesia due to hippocampal resection, such as famous patient H.M. (whose hippocampus was not completely removed) (Corkin et al., 1997), or hippocampal disease, such as patients with Alzheimer’s disease, we see an inability to retain, not so much to form, episodic memories; and the same could be said about dreaming. Hence, consciousness should not be grossly impaired in dementia, as little as it is impaired in dream states. The argument is that neural activity patterns forming through recurrent attractor dynamics in CA3 have the informational content of consciousness, in as much as they have the informational content of episodic memories *that are being formed* or *that are being recalled*; consciousness is evidence for ongoing episodic memory *formation*. Whether these memories are retained for more than seconds (during which memories are still recycled in the CA3 state space and available as after-images) is another matter. In dreaming and dementia, they are, to a large part, not. The conscious recall of episodic memories (in imagery), much like the formation of new event memories, involves attractor dynamics and pattern completion in CA3. Thus, it is not only the formation of episodic memories but also the recall of these memories that is accompanied by conscious experience. Imagery of distant autobiographical events is not impaired in most patients with dementia, as distant (long-term) memories can be *recalled*, so there is no reason to believe that the wakeful conscious experience of the external world, accompanying event memory *formation* and the recurring pattern formation by attractor dynamics in CA3 (regardless as to whether newly formed or reinstated event codes are subsequently consolidated and retained), is impaired in dementia.

While it may be true that patients with severe amnesia and deficits in spatial orientation due to extensive hippocampal damage do not show abnormal consciousness, neither do split-brain patients, unless closely examined in a neuropsychology laboratory. Would even the absence of consciousness, if no recurrent attractor dynamics were to occur in CA3, manifest itself in gross behavioral abnormalities? It is still not clear what, if anything, consciousness contributes to behavior (Zeman, 2001). The fact that we hear language or recognize faces does not mean that consciousness is critically involved in language perception or in face recognition, other than in registering these processes on a level of episodic memory, unless it could be argued that consciousness, that is, the ongoing formation of episodic (declarative) memories, contributes to self-localization and hence to the advancement of complex navigationally based behavior, so that, for instance, intermittent goal imagery during speech or thinking, being evidence for retrieval and partial reinstatement of episodic memories, would help to direct and reposition the *train* of speech or thought.

## Schizophrenic Hallucinations

Electrical stimulation of the hippocampus in animals can elicit fixation of attention to an imaginary object (discussed in Isaacson, 2002). In humans, electrical stimulation in the region of the hippocampus and amygdala (medial temporal lobe) can induce hallucinations, usually in the visual but sometimes in the auditory modality, or vivid experiences of autobiographic memories (Vignal et al., 2007). The experience of auditory verbal hallucinations (Shergill et al., 2000) or visual hallucinations (Oertel et al., 2007) by patients with schizophrenia is associated with hippocampal activation, along with activations in higher-order neocortical sensory-processing areas (as measured by functional neuroimaging), while schizophrenic patients’ *disposition* to experience hallucinations (and other positive symptoms of schizophrenia) is associated with hyperactivity of the left hippocampus (and parahippocampal gyrus) (Liddle et al., 2000). Although hippocampal volume is often reduced in patients with schizophrenia, hippocampal activity (as measured by functional neuroimaging) is often increased (Heckers, 2001). It has been hypothesized that hippocampal hyperactivity – possibly reflecting an excess of excitatory neuronal activity – and/or aberrant hippocampal firing are responsible, more or less directly, for hallucinations and other positive symptoms in schizophrenia (Liddle et al., 2000; Heckers, 2001; Olypher et al., 2006; Lodge and Grace, 2007). Excessive or disorganized firing of hippocampal neurons may be due to a deficit of inhibitory neuronal activity in the hippocampus. Postmortem studies of schizophrenia demonstrate GABAergic hypofunction in the hippocampus, especially in regions CA3 and CA2 (Benes, 2007; Benes et al., 2007). GABAergic hypofunction predominantly concerns fast-firing interneurons – such as basket cells, which contain parvalbumin and synapse onto perisomatic aspects of hippocampal pyramidal cells – given that the expression of isoenzyme GAD_67_ is reduced in postmortem hippocampal samples from schizophrenia patients (Benes et al., 2007). GAD (glutamic acid decarboxylase) synthesizes GABA in GABAergic neurons, whereby isoenzyme GAD_67_ is found especially in GABAergic neurons that fire tonically at a fast rate, such as basket cells.

Decreased functionality of fast-spiking interneurons, particularly basket cells, leads to a reduction of inhibitory postsynaptic potentials in hippocampal pyramidal neurons and compensatory upregulation of postsynaptic GABA_A_ receptors on these neurons (as can be demonstrated in CA3 and CA2 of schizophrenia patients) (discussed in Heckers et al., 2002; Gisabella et al., 2005). Decreased functionality of basket cells, in turn, may be attributable to hypofunction of glutamatergic NMDA receptors expressed on these cells. NMDA receptors on basket cells respond to extracellular glutamate levels, allowing these cells to “sense” the level of activity of surrounding pyramidal cells and to adjust their synthesis and release of GABA accordingly. If basket cells are relatively insensitive to extracellular glutamate levels, due to reduced NMDA-receptor function, GABA synthesis (by GAD_67_) in these neurons is downregulated, and less GABA would be released upon postsynaptic pyramidal cells (reviewed in Lisman et al., 2008). In patients with schizophrenia, reduced NMDA-receptor function is a consequence of genetic abnormalities, while, in healthy subjects, NMDA-receptor dysfunction can be brought about temporarily by ingestion of NMDA-receptor antagonists (reviewed in Coyle, 2006; Lisman et al., 2008). NMDA-receptor antagonists – blocking NMDA receptors on GABAergic interneurons and thereby disinhibiting hippocampal pyramidal neurons – have psychotomimetic properties, in that they can induce psychotic experiences, such as hallucinations, in healthy subjects. Moreover, NMDA-receptor antagonists can disrupt “prepulse inhibition” of the startle reflex (the ability of a weak prestimulus to reduce the magnitude of the startle response to a subsequent intense acoustic stimulus), a deficit that is commonly seen in patients with schizophrenia. In rats, prepulse inhibition was disrupted by infusion of an NMDA-receptor antagonist into the dorsal hippocampus, presumably mediated by disinhibition of hippocampal pyramidal cells (Bakshi and Geyer, 1998).

The effect of early life stresses on hippocampal integrity in patients with schizophrenia can be modeled in rats by isolation rearing. Rats reared in isolation after weaning show deficits in prepulse inhibition of the startle reflex in association with reduced density of parvalbumin- and calbindin-immunoreactive interneurons in the hippocampus, reminiscent of the combination of deficient prepulse inhibition and hippocampal GABAergic hypofunction characteristic of schizophrenia (Harte et al., 2007). Parvalbumin immunoreactivity in the hippocampus is reduced in patients with schizophrenia (as shown in postmortem samples) (Zhang and Reynolds, 2002). The density of parvalbumin-expressing basket cells may be reduced because of excessive glutamatergic neurotransmission in the hippocampus, leading to excitotoxic neuronal damage. Hippocampal basket cells produce CRH (corticotrophin-releasing hormone), which acts on CRH_1_ receptors, which are particularly densely expressed in region CA3. Life-long overexpression of CRH by basket cells, as a result of early life stresses (as shown in rats), and excessive activation of CRH_1_ receptors on CA3 pyramidal neurons enhances glutamatergic neurotransmission and promotes ongoing excitotoxic neuronal injury and neuronal loss in CA3, especially in the immature hippocampus (Brunson et al., 2001).

In another rat model of schizophrenia, excessive glutamatergic outflow from the basolateral amygdala to regions CA3 and CA2 indirectly causes excitotoxic reductions in GABAergic interneuron density, especially affecting presumptive fast-firing basket cells, mirroring postmortem findings of reduced GABAergic interneuron functionality in CA3 and CA2 in schizophrenia (Berretta et al., 2004; Gisabella et al., 2005; Benes, 2007). In humans, direct stimulation of the amygdala can elicit feelings of fear and anxiety as well as complex hallucinations. Brain lesions in or near the amygdala predispose to schizophreniform psychoses (Fudge and Emiliano, 2003). Social phobia and excessive trait anxiety are associated with hyperresponsivity of the amygdala (especially on the left) to others’ angry or contemptuous emotional expressions (as shown in neuroimaging studies) (Stein et al., 2002, 2007; Phan et al., 2006). Importantly, psychotic episodes in schizophrenia, which typically feature delusional beliefs about specific others causing harm or posing a threat to the patient, are often preceded or accompanied by syndromatic or subsyndromatic social phobia characterized by intense apprehensions, generalized across all social encounters, about being criticized, negatively evaluated, or rejected by others (Michail and Birchwood, 2009).

Whether GABAergic hypofunction in the hippocampus is caused by excitotoxic loss of fast-firing basket cells due to (i) hyperresponsivity of the amygdala and excessive glutamatergic outflow from the amygdala to the hippocampus or (ii) early life stresses and overexpression of CRH; or whether it is caused by reduced output of basket cells due to (iii) genetically determined NMDA-receptor dysfunction (Lisman et al., 2008); the result is a lack of GABA-mediated postsynaptic inhibitory potentials in hippocampal pyramidal cells. Pyramidal cells would be overexcitable, their firing would be increased, and hippocampal synaptic plasticity would be enhanced (Gisabella et al., 2005). Increased or disorganized firing of pyramidal cells would lead to the formation of event memories and conscious experiences that are underconstrained by information processed in entorhinal and upstream association neocortical regions. In other words, hallucinations may be directly attributable to excessive excitability of pyramidal neurons in CA3, which would predispose to distorted or deceptive event code generation. Alternatively, or in addition, overactive hippocampal pyramidal cells may contribute to psychosis by increasing the number of spontaneously active dopaminergic neurons in the ventral tegmental area (mediated by a multisynaptic pathway via ventral subiculum, ventral striatum, and ventral pallidum) and, thereby, driving a hyperdopaminergic state (Lodge and Grace, 2007; Lisman et al., 2008). Increased dopamine release in the ventral striatum (from terminals of dopaminergic neurons of the ventral tegmental area) may be responsible for hyperactivity, agitation, and aggressiveness associated with psychosis; in agreement with Liddle et al. (2000) who thought that, while hyperactivity in the ventral striatum seems to be “associated with acute psychosis, irrespective of symptom profile,” hyperactivity of the hippocampus is more specifically associated with “reality distortion” (delusions and hallucinations) (p. 406).

Olypher et al. (2006) suggested that disorganized firing of hippocampal pyramidal neurons provides the basis on which “a pathological steady and stable state of activity” can emerge through γ synchronization: a “parasitic attractor” “that does not reflect reality” but a “hallucination” instead (Olypher et al., 2006, p. 166). Furthermore, increased excitability of hippocampal pyramidal cells may account for impairments in Gestalt tasks that require perceptual grouping of visual stimuli – impairments that can be identified in patients with schizophrenia, especially patients with “disorganized schizophrenia” (characterized by prominent hallucinations and thought disorder) (Straube, 1975; Liddle, 1987; Nuechterlein et al., 1992). Perception entails the segregation of relevant from irrelevant stimuli, allowing selective association among relevant stimuli (“subgrouping” or “cognitive coordination”). In rats, blocking neural activity in one hippocampus (by injecting tetrodotoxin) induced coactivation, at γ frequencies, of pyramidal cells in the contralateral hippocampus that initially fired independently (Olypher et al., 2006). As a result, the ability of rats to subgroup distal spatial stimuli and segregate them from irrelevant local stimuli was impaired, preventing these rats from effectively avoiding regions of the room where footshock was administered. Coactivation at γ frequencies of initially uncoupled hippocampal pyramidal neurons prevents the segregation into cell assemblies encoding independent and unrelated representations, which would explain impairments in Gestalt perception (Olypher et al., 2006).

We need to account for the fact that hallucinations in schizophrenia are usually auditory and verbal in form, whereby “voices” heard by patients characteristically talk *about* them, as opposed to addressing them directly. There is clearly an interaction between a biological disposition to hallucinate, which can be seen in psychologically healthy individuals, too, and attentional pressures of a particular kind, the focus of which accords with the prominent role of language in the regulation of self-esteem. Language perception in patients with schizophrenia is more susceptible to psychological suggestion (Haddock et al., 1995), pointing to an excessive influence of subjective expectation on language perception. What patients with schizophrenia, who tend to be more fearful of their social environment, expect to hear – and what they therefore do hear in form of their voices – are critical and derogatory comments made by others, reflecting patients’ beliefs about their subordination, disparagement, and marginalization in society and interpersonal relationships (Linn, 1977; Nayani and David, 1996; Birchwood et al., 2000). Attentional pressures reflecting these concerns appear to outweigh peripheral sensory constraints imposed on reverberating γ-oscillatory activity in thalamocortical networks (Behrendt and Young, 2004; Behrendt, 2006). Indeed, stimulus-induced γ synchronization (Green et al., 1999; Spencer et al., 2003) – that is, entrainment, by peripheral sensory information, of intrinsic γ-frequency oscillations (Kwon et al., 1999) – in neocortical regions is impaired in patients with schizophrenia. While this may be true for paranoid schizophrenia, excessive or disorganized hippocampal activity may be the predominant pathophysiological mechanism of hallucinations in disorganized or hebephrenic schizophrenia, the clinical picture of which features visual, alongside auditory, hallucinations as well as prevalent formal thought disorder.

## Conclusion

The assignment of a critical role in the generation of conscious perception – and, ultimately, of conscious experiences of all kinds – to the hippocampus arises from the following considerations. Firstly, conscious perception is intricately linked with the formation of episodic (declarative) memories, much as conscious imagery is linked with episodic memory retrieval. Secondly, conscious perception is allocentric. What we experience consciously is a world that surrounds us and is independent of our observation, as is evident when considering the phenomenology of conscious perception and dreaming, from which more fleeting and seemingly internal types of conscious experience are likely derived. What is characteristic of conscious perception and dreaming alike is that objects or events are experienced as being embedded in an external, that is, allocentric, frame of space and time. The hippocampus has been implicated in the rapid formation and memorization of allocentric representations that embed objects or events in a world context. Thirdly, the hippocampus is ideally placed to bind information processed in different sensory-processing streams. Object-related and emotional information from the lateral entorhinal cortex and spatial contextual information from the medial entorhinal cortex are combined by hippocampal region CA3 into unique event memory codes or indices (Morris et al., 2003; Kesner, 2007; Rolls, 2007). Rapidly forming patterns of neuronal ensemble firing in hippocampal region CA3, which encode arbitrary associations between objects and their spatiotemporal and emotional context, that is, associations between information derived from different neocortical processing streams, may define the informational content of consciousness. Conscious experience would be a signature or symbolic representation of the pattern of activity emerging in CA3 through self-organizing attractor dynamics.

Conscious experience of a substantial world that seems to surround us is *episodic memory* *formation* in action; conscious imagery of a less substantial, seemingly internal world is *episodic memory* *retrieval* in action. Every recall (in imagery) of a previous experience (memory trace of full consciousness) is evidence for the retrieval of an episodic memory. The ability of the episodic memory system to extract and recombine elements of previous experiences can be utilized for the purpose of decision making and prospective guidance of behavior (goal imagery, outcome simulation). The hippocampus may bind features of novel scenarios for the purpose of simulating and anticipating future outcomes, similarly to how it binds features during the retrieval and conscious elaboration of past autobiographical events (Addis et al., 2007; Schacter and Addis, 2007). Outcome simulation (goal imagery) is supported by competitive activations of the default-mode network during the performance of complex tasks (the pursuit of hierarchically organized behavioral objectives). Internal mentation, too, draws on the ability of the episodic memory system to extract and recombine elements of previous experiences; and it, too, involves the default-mode network. For as long as the subject is not engaged in the performance of novel or demanding tasks, and while focused attention is not required, the default-mode network is engaged in gathering and evaluation of information “broadly arising in the external and internal milieu” (Raichle et al., 2001, p. 682). The default-mode network may support monitoring or observation of the external milieu, on the one hand, and states of internal mentation and episodic memory recollection, on the other, precisely insofar as it is functionally coupled with the hippocampus, the critical node, as it is suggested, in all conscious processes.

Conscious memory recall or outcome simulation (in imagery) may be attributable to retrieval cues (encoded in neocortical areas) inducing the reinstatement of neuronal assemblies in CA3 via direct entorhinal input to CA3. Conscious recall of distant autobiographical memories is mostly intact in amnesic patients with dementia (including those with Alzheimer’s disease whose hippocampus undergoes neurodegeneration early in the disease process), so that it not surprising that these patients are able retain new *declarative* information for seconds at least, if not minutes, and that their consciousness is generally not impaired. What is impaired in dementia is not so much the ability to form new episodic (declarative) memories or to recall past memories, which suggests that rhythmic pattern formation in CA3 still does occur, but the ability to consolidate new episodic memories. Similarly, dreaming is evidence for ongoing episodic memory *formation*, yet there is hardly any retention. Only small fragments of dream experiences can later be recalled, based on very limited reinstatement of event codes that were previously fully formed during the dream experience. Increased activity in the CA3-dentate network (Montgomery et al., 2008), while the default-mode network is inactive, has been associated with REM sleep and may be responsible for the dreaming experience. Evidence for GABAergic deficit and pyramidal cell hyperexcitability in CA3 in patients with schizophrenia is consistent with the notion that binding, by the CA3 network, of weakly related representations underlies hallucinations in this disorder (Olypher et al., 2006).

## Conflict of Interest Statement

The authors declare that the research was conducted in the absence of any commercial or financial relationships that could be construed as a potential conflict of interest.
